# Malignant Peripheral Nerve Sheath Tumor of the Inguinum and Angiosarcoma of the Scalp in a Child with Neurofibromatosis Type 1

**DOI:** 10.1155/2017/7542825

**Published:** 2017-08-24

**Authors:** Marija Milković Periša, Tihana Džombeta, Jasminka Stepan Giljević, Božo Krušlin

**Affiliations:** ^1^Institute of Pathology, School of Medicine, University of Zagreb, Šalata 10, 10000 Zagreb, Croatia; ^2^Department of Pathology, Clinical Hospital for Tumors, University Clinical Hospital Centre “Sestre Milosrdnice”, Ilica 197, 10000 Zagreb, Croatia; ^3^Department of Pathology, University Clinical Hospital Centre “Sestre Milosrdnice”, Vinogradska 29, 10000 Zagreb, Croatia; ^4^Department of Oncology and Hematology “Mladen Ćepulić”, Children's Hospital Zagreb, Klaićeva 16, 10000 Zagreb, Croatia

## Abstract

Benign and malignant tumors are common in the setting of neurofibromatosis type 1 (NF1). Malignant peripheral nerve sheath tumor (MPNST) and angiosarcoma are rare tumors in children and adolescents and mostly occur in young patients in relation to NF1. Both histological types can be present in the same tumor mass in patients with NF1. We present a case of 12.5-year-old girl with NF1 who first presented with MPNST of the right inguinal region and 1.5 years later with unrelated angiosarcoma of the scalp.

## 1. Introduction

Neurofibromatosis type 1 (NF1) is a common autosomal dominant disorder with the incidence rate of 1 in every 3500 individuals [1]. The main characteristics of this disorder are “café-au-lait” spots, cutaneous neurofibromas, and hamartomas of the iris (Lisch nodules) [1]. Affected individuals have increased risk of developing benign and malignant tumors which include neurofibromas, gliomas, malignant peripheral nerve sheath tumors (MPNSTs), and nonneural tumors like pheochromocytoma, myelogenous leukemia, and multifocal gastrointestinal stromal tumors [1, 2]. Malignant peripheral nerve sheath tumors and angiosarcomas are uncommon in children and adolescents, even those suffering from NF1. Some authors described these two entities within the same tumor mass, since heterologous differentiation in the form of angiosarcoma may occur in MPNST, more commonly in patients with NF1 [3–5]. Here, we report a case of MPNST of the inguinal region and unrelated angiosarcoma of the scalp in a girl with NF1.

## 2. Case Report

**Figure 1:**
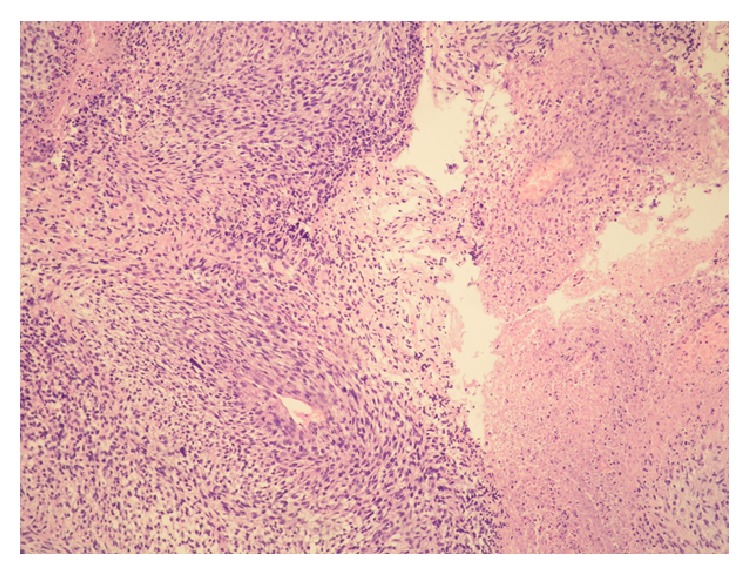
Malignant peripheral nerve sheath tumor histologically showing fascicles of spindle cells (left) and necrosis (right) (HE, ×100).

A 12.5-year-old girl with a history of neurofibromatosis type 1 presented in January 2015 with a right inguinal tumor mass causing pain and impairment of movement of the right leg. Magnetic resonance imaging showed a heterogeneous solid-cystic tumor mass with focal necrosis, measuring 9.6 cm in the greatest dimension. Tumor biopsy was performed. Histologically, the material comprised focally necrotic tumor fragments, composed of atypical spindle to polygonal cells showing high mitotic activity, forming fascicles with focal palisading (Figure 1). Immunohistochemically, tumor cells were positive for S-100 and negative for desmin. Considering the aforementioned, the case was signed out as malignant peripheral nerve sheath tumor. The girl was treated with two cycles of chemotherapy according to the CWS protocol and one cycle of vinblastine. The control imaging scans showed an evident progression of the tumor, which was then surgically excised in June 2015. The resected tumor was histologically completely necrotic; thus the previous pathohistological diagnosis could not be confirmed.

**Figure 2:**
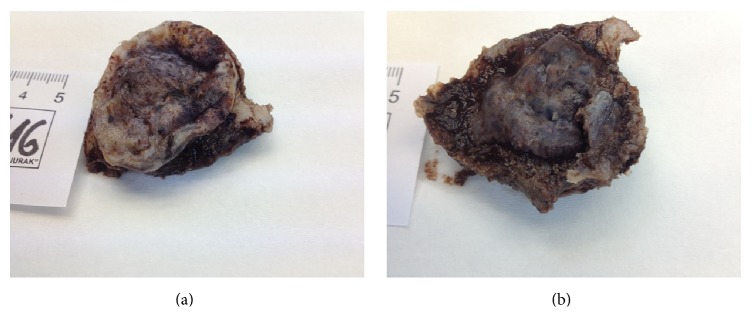
Angiosarcoma of the scalp. A gross appearance of the resected specimen, as seen from the skin surface (a) and epidural surface (b).

**Figure 3:**
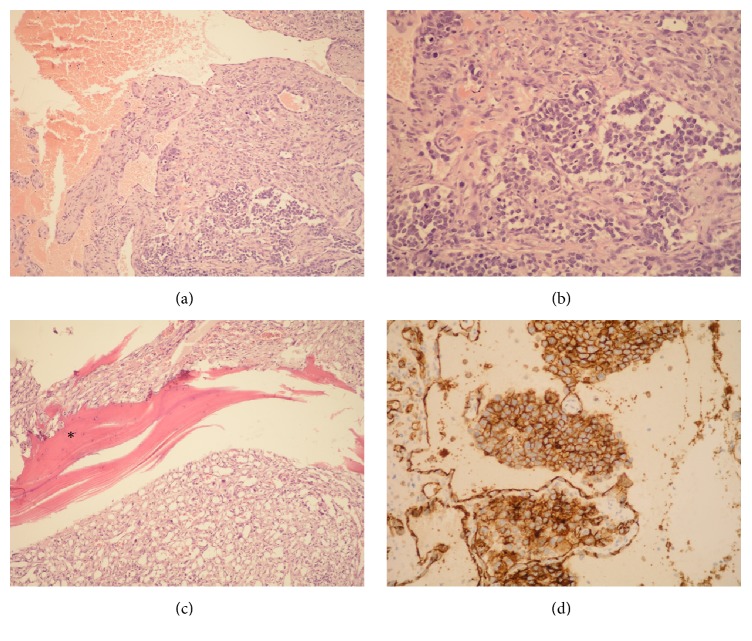
Angiosarcoma of the scalp. Histologically, the tumor was composed of aberrant vascular spaces, covered with atypical endothelial cells showing focal proliferation in the form of intraluminal buds ((a) HE, ×100; (b) HE, ×200). The tumor infiltrated the bone (asterisk) ((c) HE, ×100). Tumor cells showing immunohistochemical expression of CD31 ((d) ×200).

One year later, in June 2016, after falling down the stairs, a painful lesion of the right parietal region was found. Ultrasound and CT scans showed a soft tissue tumor measuring 3 cm in the greatest diameter, which was penetrating through the skull into epidural space. In July 2016 the tumor was surgically resected (Figures 2(a) and 2(b)). Histologically, this tumor was composed of aberrant vascular, focally anastomosing spaces covered with atypical endothelial cells showing prominent nucleoli and high mitotic activity (Figures 3(a) and 3(b)). Tumor infiltrated through the bone (Figure 3(c)). Immunohistochemically, the tumor cells were diffusely positive for CD31 (Figure 3(d)), CD34, and Factor VIII. The pathohistological findings were consistent with the diagnosis of angiosarcoma. This lesion of the scalp did not contain any spindle cell areas which could correspond to MPNST nor did it show connection to any peripheral nerve; therefore it was considered a de novo lesion, unrelated to previously diagnosed and treated MPNST of the right inguinal region.

In September 2016 the patient presented with recurrent MPNST of the right inguinum, measuring 2,5 cm, which led to the reintroduction of chemotherapy according to CWS protocol. After the third cycle of chemotherapy, in December 2016, a MR scan was performed, showing no evidence of previously described tumor mass.

In March 2017, 26 months after the initial diagnosis of MPNST of the inguinum and 9 months after the diagnosis of angiosarcoma of the scalp, the patient had no evidence of tumor masses. She is under the regular follow-up care.

## 3. Discussion

The prevalence of MPNST in NF1 patients is 4,6%, which yields a 4600 times greater risk than that of general population [6]. In these patients MPNSTs mostly occur as malignant transformation of the preexisting plexiform neurofibromas [7]. However, even in children suffering from NF1, MPNST is rare, since the greatest risk for developing sarcoma in NF1 patients is 10 to 20 years after the appearance of neurofibromas, at the age of 20 to 50 years [7]. In NF1 patients, MPNSTs usually have characteristic histological appearance showing densely packed fascicles of spindle cells with wavy nuclei and malignant features, such as high mitotic activity, necrosis, and evident cell atypia. All of these were also present in the inguinal tumor mass found in our patient. Patients with NF1 have an increased risk of developing various benign and malignant tumors and among these are also angiosarcomas, but usually as a part of MPNST [4, 5]. Brown et al. [5] described a case of a 20-year-old male with NF1 who developed angiosarcoma arising from a MPNST. The authors reviewed previously reported cases of angiosarcoma in patients with NF1 and discovered that 50% of them (4/8) were MPNST related, meaning they arouse as a sarcomatous transformation of a previously existing neurofibroma, and most of the others were associated with a peripheral nerve. Three of them were localized in a cervical brachial plexus, one in scrotum, femoral nerve, radial nerve, and liver, and one was of unknown primary site. All of the patients were male. Only one tumor, occurring in a 61-year-old male with NF1, localized in the scrotum, was not connected to a nerve, nor did it have a MPNST component [5]. Elli et al. [4] described a case of a 13-year-old boy with NF1 who presented with intrathoracic MPNST with angiosarcoma. None of the reported cases of angiosarcoma in NF-1 patients was reported in the scalp. Cutaneous primary angiosarcoma usually develops on the face or scalp and predominantly affects elderly people, frequently men [8–10]. Choi et al. [11] reported a retrospective analysis of 14 patients with angiosarcoma of the scalp and Mullins and Hackman [12] reported 5 more patients. Most of the patients in both of these studies were men, with only one woman, while the mean age was 69 and 65.5 years. Both of the studies concluded that this malignancy is very aggressive, with prognosis dependent upon size and completeness of tumor excision [11, 12]. The only case of an angiosarcoma of the scalp occurring in a pediatric patient that we found was the one described by Khan et al. [13]. Angiosarcomas are very rare in pediatric population, although they sometimes occur in patients with NF1, but then they are usually MPNST related. Therefore, we believe that the case of our patient, a girl with NF1 who at the age of 14 developed an angiosarcoma of the scalp unrelated to benign or malignant nerve tumor, is exceptionally rare.
